# Combined Minimal Residual Disease Evaluation in Bone Marrow and Apheresis Samples in Multiple Myeloma Patients Undergoing Autologous Stem Cell Transplantation Improves Outcome Prediction

**DOI:** 10.3390/cancers17152439

**Published:** 2025-07-23

**Authors:** Irene Attucci, Benedetta Peruzzi, Chiara Nozzoli, Serena Guerrieri, Sofia Pilerci, Riccardo Boncompagni, Serena Urbani, Chiara Orazzini, Sara Bencini, Manuela Capone, Maria Messeri, Roberto Caporale, Francesco Annunziato, Alessandro M. Vannucchi, Elisabetta Antonioli

**Affiliations:** 1 Haematology Unit, Careggi University Hospital, Largo Brambilla 3, 50134 Florence, Italymaria.messeri@unifi.it (M.M.);; 2 Flow Cytometry and Immunology Diagnostic Unit, Careggi University Hospital, Largo Brambilla 3, 50134 Florence, Italyorazzini.chiara@gmail.com (C.O.);; 3 Cell Therapy and Transfusion Medicine Unit, Careggi University Hospital, Largo Brambilla 3, 50134 Florence, Italy; 4 Department of Experimental and Clinical Medicine, University of Florence, Largo Brambilla 3, 50134 Florence, Italy; serena.guerrieri.sg@gmail.com; 5 USL Toscana Nord Ovest, Versilia Hospital, Via Aurelia n. 335, 55041 Lido di Camaiore, Italy; 6 Immunohematology and Transfusion Medicine Unit, S. Stefano Hospital, Via Suor Niccolina Infermiera 20/22, 59100 Prato, Italy

**Keywords:** minimal residual disease (MRD), apheresis samples, multiple myeloma

## Abstract

**Introduction:** Despite the approval of novel agents that have significantly improved long-term survival rates for multiple myeloma (MM) patients undergoing autologous stem cell transplant (ASCT), most patients eventually relapse. The failure to achieve or maintain bone marrow (BM) minimal residual disease (MRD) negativity is a recognised adverse prognostic factor for progression-free survival (PFS) and overall survival (OS). Contamination of stem cell apheresis by clonal plasma cells may also affect prognosis, though data remain limited. **Methods:** We conducted a prospective, single-centre observational study including 100 newly diagnosed MM patients eligible for ASCT and treated with bortezomib-based triplet induction. MRD was assessed both on BM and apheresis samples using multiparameter flow cytometry (MFC-MRD) with a sensitivity of 10^−5^. **Results:** Clonal plasma cells were detected in 22 apheresis samples (aMRD+), all of which were associated with BM MRD positivity. Patients with aMRD+ had inferior pre-ASCT responses (≥VGPR: 10% vs. 63%, *p* = 0.005) and worse post-ASCT BM MRD negativity rates (4% vs. 49%, *p* = 0.048). After a median follow-up of 52.4 months, aMRD+ was associated with shorter progression-free survival (median 38.5 vs. not reached, *p* = 0.007) and overall survival (median 60 months vs. not reached, *p* = 0.003). **Conclusions:** Contamination of the apheresis product is associated with persistent BM disease and poorer outcomes. Combined MRD assessment in both bone marrow and apheresis may improve risk stratification in MM patients undergoing ASCT.

## Simple Summary

Despite advances in treatment, most multiple myeloma patients undergoing autologous stem cell transplant (ASCT) eventually relapse. It is well established that the failure to achieve or maintain bone marrow minimal residual disease (MRD) negativity is associated with reduced progression-free survival and overall survival. In this prospective study, we evaluated the prognostic value of MRD assessment not only in bone marrow but also in apheresis samples. The presence of clonal plasma cells in the apheresis sample was consistently associated with poorer responses to induction therapy and lower rates of post-transplant bone marrow MRD negativity. These patients also showed significantly reduced survival outcomes. Our findings suggest that evaluating MRD in both bone marrow and apheresis samples provides a more comprehensive risk assessment and may enhance outcome prediction in multiple myeloma patients undergoing ASCT.

## 1. Introduction

The therapeutic landscape of multiple myeloma (MM) [1] is continually evolving, and highly effective triplet or quadruplet regimens are now used in earlier lines of treatment [2], leading to significant improvements in progression-free survival (PFS) and overall survival (OS) [3]. Autologous stem cell transplantation (ASCT) remains the standard of care for newly diagnosed transplant-eligible MM patients, as outlined by the guidelines from the European Haematology Association (EHA) and the European Society for Medical Oncology (ESMO) [4]. Recent phase III trials comparing the use versus omission of upfront ASCT following induction with triplet novel agents demonstrated improved PFS in the upfront ASCT arm [5]. Additionally, a three-drug combination, including at least bortezomib and dexamethasone (such as VTD, or, where approved, the VRD combination), has become the standard of care when a quadruplet regimen with an anti-CD38 antibody is not available [6]. However, despite the incorporation of novel agents significantly improving long-term survival rates for MM patients undergoing ASCT, most patients experience relapses at various times post-ASCT. Contamination of stem cell grafts by abnormal plasma cells (APCs) has been considered a potential predictor of subsequent clinical outcomes, but several small-scale studies have produced conflicting findings [7,8]. Almost all of these studies have reported a link between residual plasma cells in the apheresis product after induction therapy and reduced rates of complete responses (CR) [9,10,11]. Pasvolsky et al. conducted an extensive analysis involving 416 high-risk multiple myeloma patients and found a correlation between graft contamination and decreased levels of minimal residual disease (MRD) negativity in bone marrow (BM), as well as poorer PFS and OS [10]. However, other studies have produced inconsistent results regarding the impact of APCs on PFS and OS [7,9,11,12,13,14]. The aim of our study is to assess the impact of autograft contamination by APCs in newly diagnosed MM patients undergoing ASCT. We investigated the efficacy of minimal residual disease assessment using multiparameter flow cytometry (MFC-MRD) in both pre-transplant and post-transplant bone marrow and apheresis samples. Our study prospectively analysed the influence of MRD on overall response rate (ORR), PFS and OS in a cohort of uniformly treated consecutive patients.

## 2. Methods

This single-centre prospective observational study was conducted between June 2017 and August 2023 at the Haematology and Cell Therapy Unit of Careggi Hospital in Florence. As inclusion criteria, we considered patients with newly diagnosed MM who were eligible for ASCT and treated at our centre. Procedures involving donors’ specimens were approved by institutional ethics committees and conducted in accordance with the principles of the Declaration of Helsinki. All patients provided written informed consent at enrolment.

**Figure 1:**
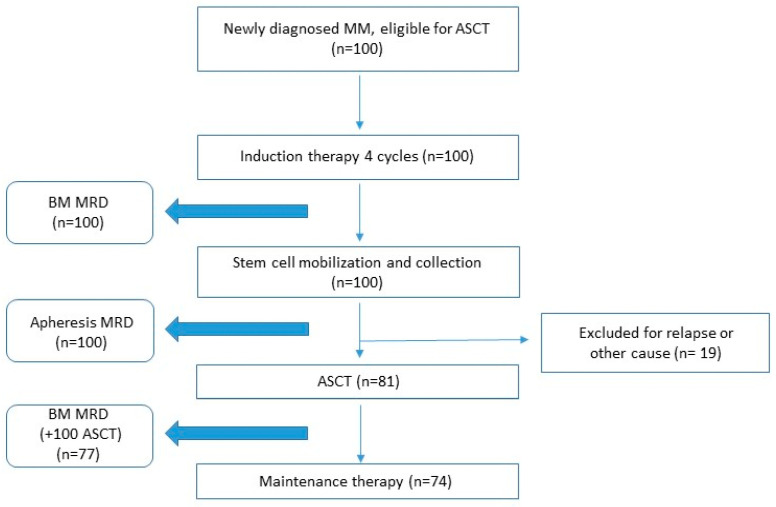
Timeline. ASCT = autologous stem cell transplantation; BM = bone marrow; MRD = minimal residual disease; MM = multiple myeloma.

One hundred (100) patients were enrolled, presenting a diagnosis of symptomatic disease according to the International Myeloma Working Group (IMWG) criteria [15]. Baseline data at diagnosis were collected for each patient. For risk stratification, we used the International Staging System (ISS) [16] and Revised-ISS [17]. We defined high-risk cytogenetic features as the presence of any one of the following abnormalities: del(17p), t(4;14), t(14;16), t(14;20), or amplification(1q). These were analysed by fluorescence in situ hybridization (FISH) in CD138+ plasma cells using standard methodology [18]. All patients received bortezomib-based induction treatment. Disease response was assessed through blood and urine tests after each cycle, according to IMWG response criteria [19]. The achievement of at least a partial response (PR) after induction was required for the completion of the autologous stem cell transplantation-based therapeutic plan. Peripheral blood stem cell (PBSC) mobilisation and collection were performed with cyclophosphamide (CY) (from 2 to 4 gr/m^2^) plus filgrastim (10 µg/Kg/day) or filgrastim alone according to local standard protocols. Plerixafor was used to overcome poor stem cell mobilisation. The optimal apheresis target dose was 4 × 10^6^ CD34+/kg. All patients underwent PBSC collection with the Spectra Optia© apheresis system. All patients underwent conditioning therapy with melphalan 200 mg/m^2^ (MEL200), followed by the reinfusion of PBSC the day after. The post-ASCT response was assessed 3 months (+100 days) after transplantation through complete blood and urine tests. Bone marrow evaluations and MFC-MRD measurements were performed after induction cycles and on day +100 post-transplant in all patients who achieved at least a very good partial response (VGPR) (Figure 1). Consolidation treatment was not administered, while lenalidomide monotherapy as maintenance treatment was recommended until progression or unacceptable toxicity [20].

### 2.1. Flow Cytometry Assessment of MRD Status in Bone Marrow and Apheresis Sample

The presence of APCs in BM samples and PBSC was assessed using an 8-colour FC panel for MM evaluation, achieving a sensitivity threshold of 10^−5^. This panel included the following antibody combinations: CD38-FITC, CD56-PE, CD45-PerCPCy5.5, CD19-PE-Cy7, CD117-APC, CD81-APCH7, CD138-V450 and CD27-BV500. For MRD assessment, a CD38 multiepitope was added to the mixture of antibodies. The white blood cell count of EDTA-anticoagulated BM aspirate or apheresis sample was performed using an XN 550 haematology analyser (Sysmex Corporation, Kobe, Japan). A sample volume corresponding to 5 × 10^6^/mL white blood cells was labelled according to the 8-colour panel for MM. Cells were incubated with the mixture of monoclonal antibodies for 15 min at room temperature in the dark. After staining, cells were lysed to remove red blood cells and washed with FC buffer before they were acquired on the flow cytometer FACSCanto II (BD Biosciences, San Jose, CA, USA), which was quality-controlled on a daily basis using CS&T Beads (BD Biosciences) [21]. Acquired data were analysed using the Infinicyt software (version 2.1) (Cytognos S.L., Salamanca, Spain).

Cells were gated using the combination of CD38, CD138, CD45 and side scatter (SSC) and followed the gating strategy as described in the literature [22]. To allow accurate MFC-MRD assessment at the limit of detection (LOD) and limit of quantification (LOQ), a minimum denominator of at least 3,000,000 CD45 expressing events was acquired, as recommended by IMWG consensus criteria [19]. Recording at least 3 million events leads to a minimum of LOD 0.001% and LOQ 0.0017%, estimated as (30/total cells analysed) × 100% and (50/total cells analysed) × 100%, respectively [23]. MRD status was considered MRD positive when ≥50 plasma cells (LOD) with an abnormal phenotype were identified from the total nucleated compartment.

### 2.2. Statistical Analysis

All analyses were on an intent-to-treat basis. Statistical analysis was performed using the IBM SPSS Statistics for Windows (Version 29.0, Armonk, NY, USA) and R (version 4.5.1) software. For all continuous variables, the median and interquartile range (IQR) were reported. Categorical variables were summarised using absolute frequencies and percentages. Comparisons between the aMRD+ and aMRD− groups were performed using Fisher’s exact test. PFS was calculated from the start date of induction therapy to the date of disease progression, death (in the absence of progression), or last follow-up. Patients who were alive without documented disease progression at the time of last follow-up were censored. OS was measured from the date of start of induction treatment to the date of last known vital status, with patients still alive at last follow-up being censored. OS and PFS were estimated using the Kaplan–Meier method, and group differences were evaluated with the log-rank test. Associations between clinical outcomes and variables of interest were analysed using both univariate and multivariable Cox proportional hazards regression models. A *p* value of less than 0.05 was considered to indicate statistical significance.

## 3. Results

### 3.1. Patients

From July 2017 to November 2021, bone marrow and apheresis samples from 100 consecutive, unselected patients with newly diagnosed multiple myeloma, who were eligible for autologous transplant, were analysed. Baseline characteristics are summarised in Table 1. The median age was 59 years (range 37–70), and 51% of patients were male. Twenty-six patients (26%) had high-risk cytogenetic abnormalities at diagnosis.

All patients received four cycles of induction treatment with bortezomib-based triplet regimens (VTD 96%, PAD 4%). The overall response rate (≥PR) after induction therapy was 96%, with 73% of patients achieving a VGPR or better. All 100 patients underwent successful stem cell mobilisation after induction treatment. PBSC collection was carried out with cyclophosphamide and filgrastim in 90% (90/100) of cases, while 10% (10/100) of patients underwent stem cell collection following filgrastim stimulation alone. The median follow-up for the overall cohort was 52.7 months; the most recent data cut-off was 20 December 2024.

Among the entire cohort, 19 (19%) patients initially intended to be transplanted, but could not proceed to ASCT, mainly due to disease progression (73%) or infectious complications and loss of fitness (27%) (Figure 1). Considering the 81 patients who underwent ASCT, the overall response rate at day +100 after transplant was 100%. To be specific, CR and sCR were 45 (55%) and 15 (19%), respectively, VGPR were 18 (22%) and PR were 3 (4%). After ASCT, 74 patients (80%) received lenalidomide maintenance. The median follow-up period was 48.3 months (range 4.4–80.6 months). Within this monitoring period, 26 patients (32%) showed evidence of disease progression, and 8 (10%) patients died, but 50% were due to an unrelated cause.

### 3.2. Bone Marrow and Apheresis Sample MRD Results and Phenotypic Features of APCs

MFC-MRD analysis post-induction was performed on bone marrow samples from all 100 patients, with 62 patients (62%) testing positive and classified as “bmMRD+”. The same MRD analysis conducted on apheresis samples revealed the presence of APCs in 22% (22/100) of patients, who were classified as “aMRD+”. All 22 aMRD+ patients also exhibited concurrent persistence of bmMRD+ at the post-induction time point. On the other hand, 40 patients (51%) without APCs in the apheresis (aMRD−) showed persistence of disease on bone marrow (bmMRD+). None of the patients with negative bmMRD (bmMRD−) had monoclonal plasma cells in the apheresis sample, showing a statistically significant correlation between persistence of bone marrow MRD after induction treatment and the presence of monoclonal plasma cells in the graft (*p* < 0.001). After ASCT, bone marrow samples from 77 out of 81 patients were available for MFC-MRD analysis at the second time point. On day +100 post-transplant, 26% of patients remained bmMRD+, while 29% showed a conversion from bmMRD+ to bmMRD−. All pre-transplant bmMRD− patients remained negative at the second time point. The overall post-transplant bmMRD negativity rate was 74% (57/77 pts).

We also evaluated the potential impact of the mobilisation strategy (cyclophosphamide plus G-CSF vs. G-CSF alone) on the apheresis product. In patients whose PBSC collection was performed using G-CSF alone, we did not observe a higher frequency of graft contamination: 1/10 were aMRD+ among the G-CSF alone cohort compared to 21/90 aMRD+ patients in the cyclophosphamide plus G-CSF group (*p* = 0.3).

In patients with aMRD+, the phenotype of APCs in the stem cell graft closely resembled the expression pattern of surface molecules identified in bone marrow samples at diagnosis. Specifically, APCs in the graft were negative for CD19, CD45 and CD81 in 100%, 86% and 54% of cases, respectively. Positivity for CD56 and CD117 was found in 68% and 50% of the aMRD+ samples, while CD27 was either negative or only weakly expressed in 41% of them.

### 3.3. Outcome and Correlation with PFS and OS

**Figure 2:**
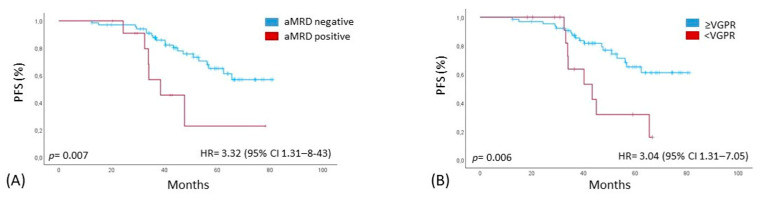
(**A,B**) PFS, Kaplan–Meier curve of progression-free survival (PFS) comparing aMRD− and aMRD+ patients in all patients and patients based on response of induction treatment. aMRD = minimal residual disease on stem cell apheresis sample.

The presence of APC contamination in the apheresis samples showed no correlation with the patients’ baseline clinical or prognostic characteristics, including sex, type of monoclonal component, ISS/R-ISS stage and cytogenetic risk. In all patients that achieved a CR/sCR after induction, no graft contamination was identified; instead, 19% of patients who achieved a VGPR were aMRD+. Among patients who achieved a PR or less (27%), 44% showed APC contamination. Only 10% of patients with at least a VGPR had APC contamination, compared to 63% of those with a less deep response (*p* = 0.005), demonstrating that the frequency of contamination in the stem cell graft was significantly associated with the depth of response to induction therapy. Therefore, we focused our subsequent analysis on 81 patients who underwent ASCT. The ORR at day +100 after transplant was 100%; 50 patients improved their response after the transplantation procedure; 10 in the aMRD+ group and 40 in the aMRD− group. However, unlike previous observations, we did not find a significant correlation between response after ASCT and aMRD status. In 77/81 (95%) patients, flow cytometric analysis on bone marrow samples was repeated at day +100 after transplant. Twenty patients (26%) showed a MRD-positive (bmMRD+). On the other hand, 57 patients (74%) achieved bone marrow MRD negativity post-transplant. A significant correlation between the achievement of at least a VGPR post-induction and bmMRD negativity post-ASCT was identified: 51/63 of the patients (63%) in ≥VGPR post-induction obtained bmMRD negativity post-transplant vs. 6/14 of the patients (7.4%) who had less than VGPR pre-ASCT (*p* = 0.006). Moreover, a significant association was documented between aMRD+ and the persistence of bmMRD positivity after ASCT: 26% of bmMRD+ patients were also aMRD+ (*p* = 0.03), confirming the difficulty of achieving bone marrow MRD negativity after the transplant in patients with contamination from monoclonal plasma cells in the apheresis sample. After a median follow-up of 52.4 months (range 18–81 months), the median PFS of all cohort was not reached, with a 48-month PFS of 76%. Considering the impact of APCs in apheresis samples, the median PFS was 38.5 months in the aMRD+ group compared to not reached in aMRD− patients [*p* = 0.007, HR 3.32 (95% CI 1.31–8.43)] (Figure 2A). In addition, we analysed with a univariate analysis the impact on PFS of the following factors: cytogenetic risk and ISS stage at diagnosis, response ≥VGPR after induction, bmMRD+ before and after transplant and achievement of at least a CR post-ASCT. Besides the presence of monoclonal plasma cells in the apheresis sample, the only other factor with a significant impact on PFS was achieving at least a VGPR before the autologous transplant [43.5 months vs. NR, *p* = 0.006, HR 3.04 (95% CI 1.31–7.05)] (Figure 2B).

None of these parameters remained statistically significant in the multivariate analysis. Moreover, the presence of APCs in the apheresis sample significantly affects PFS, which is lower across all patient subgroups, regardless of the response achieved either before or after ASCT (Table 2). Specifically, aMRD+ patients with less than a VGPR after induction had a significantly shorter median PFS compared to aMRD− patients with the same response (33.9 vs. 45.1 months), and even more so when compared to patients who achieved at least a VGPR and were aMRD− (33.9 months vs. NR, *p* < 0.001). Similar results were observed when comparing aMRD status with response after transplant: aMRD+ and a response less than CR were associated with a lower median PFS compared to aMRD− patients who achieved a CR or better (33.9 months vs. NR, *p* < 0.001). Furthermore, we evaluated the impact on PFS of bmMRD pre- and post-transplant in association with the presence or absence of APCs in stem cell apheresis (Table 2). The cohort with MRD+ in both the bone marrow and apheresis before ASCT had a shorter PFS (38.6 months vs. NR, *p* = 0.025), as did patients with a contaminated apheresis sample and persistent bmMRD positivity post-transplant, showing a median PFS of 35 months (vs. NR; *p* < 0.001).

**Figure 3:**
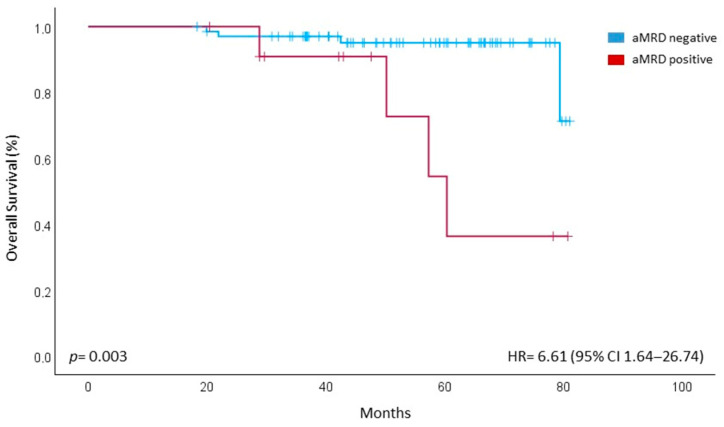
OS, Kaplan–Meier curve of overall survival (OS) comparing aMRD− and aMRD+ patients. aMRD = minimal residual disease on stem cell apheresis sample.

The median OS was not reached for the entire cohort, with a 48-month OS of 93%. However, patients with aMRD+ were associated with reduced OS compared to those without APCs contamination in the apheresis sample [60 months vs. NR, *p* = 0.003, HR = 6.61 (95% CI 1.64–26.74)] (Figure 3). None of the other factors analysed in the univariate analysis (cytogenetic risk, ISS/R-ISS stage, response ≥ VGPR before transplant or CR after ASCT, bmMRD status before and after transplant) had any impact on OS.

Furthermore, apheresis contamination with APCs and achieving less than VGPR after induction were associated with a shorter median overall survival (57 months vs. NR, *p* < 0.001; Table 3). Additionally, failure to achieve a CR post-transplant in patients with aMRD+ was linked to poorer OS (58 months vs. NR, *p* < 0.001). Moreover, the persistence of bmMRD+ both pre- and post-transplant, particularly in patients with a contaminated graft, was associated with a worse median OS (60 months vs. NR, *p* = 0.01), as shown in Table 3.

## 4. Discussion

Several studies and meta-analyses have established the prognostic value of MRD, currently considered a surrogate marker for PFS and recently proposed as a potential endpoint for clinical trials [24,25,26,27]. Bone marrow MRD negativity has been associated with significantly improved survival outcomes, regardless of disease setting (whether newly diagnosed or relapsed/refractory MM), MRD sensitivity thresholds, cytogenetic risk, method of MRD assessment, or the timing of MRD measurement [28]. Different studies revealed that the optimal MRD level associated with the best outcomes is below 10^−6^ [29], although in clinical practice, assays that have a sensitivity of 10^−5^ are considered equally informative [30], at least for patients with standard cytogenetic risk. Moreover, a significant biological and clinical concordance between flow cytometry (MFC or NGF) and NGS analysis at the same sensitivity level suggests their possible use in MRD evaluation [28,31]. Bone marrow remains the first choice for MRD detection to date; nevertheless, some studies have highlighted the prognostic value of MRD assessment on extramedullary compartments, such as the apheresis samples in transplant-eligible patients. In our study, we prospectively assessed the prognostic impact of MRD in apheresis samples from a large cohort of newly diagnosed MM patients treated uniformly using the sensitive second-generation MFC approach. Additionally, we cross-referenced the data with simultaneous bone marrow MFC-MRD evaluations performed at various time points. We detected APCs in 22% of apheresis samples at different levels, and our findings closely align with Pasvolski’s results [10], who reported 18% contamination, but differ significantly from Kostopoulos’ study [11], which found graft contamination in approximately 40% of cases. Unlike previous studies, our patients were uniformly treated according to the VTD regimen for induction and in the post-ASCT maintenance. The phenotype of APCs in grafts showed no differences compared to those detected at baseline, representing residual cells from the major aberrant population observed at diagnosis, and the presence of graft contamination did not correlate with patients’ baseline characteristics. Notably, all aMRD+ patients also exhibited persistent BM MRD positivity (bmMRD+) after induction therapy. This strong correlation between aMRD+ and bmMRD+ at the pre-transplant stage (*p* < 0.001) suggests that apheresis contamination is reflective of persistent disease burden despite induction therapy. Moreover, our findings indicate that achieving a deeper response post-induction was associated with a lower likelihood of stem cell contamination, as only 10% of patients with at least a very good partial response (VGPR) had APCs contamination, compared to 63% of those with a partial response (PR) or less (*p* = 0.005). Despite the ORR at day +100 post-ASCT being 100%, with 74% of patients achieving BM MRD negativity, a significant association was found between aMRD+ status and persistent BM MRD positivity after ASCT (*p* = 0.03). This suggests that graft contamination may hinder the ability to achieve bone marrow MRD negativity post-transplant, which is known to be a strong predictor of improved long-term outcomes. Importantly, PFS analysis revealed a clear impact of apheresis contamination on disease progression. The median PFS was significantly lower in the aMRD+ group (38.5 months) compared to the aMRD− group, where the median PFS was not reached (*p* = 0.007). This trend persisted across all subgroups, regardless of pre- or post-transplant response. Specifically, aMRD+ patients with <VGPR post-induction had the worst median PFS (33.9 months), whereas those achieving at least VGPR and aMRD− had superior PFS (NR, *p* < 0.001). The presence of APCs in the apheresis sample was also associated with inferior PFS in patients with persistence of bone marrow MRD+ after ASCT, further reinforcing its prognostic significance (*p* < 0.001). Interestingly, besides the apheresis sample contamination, the only other prognostic factor that showed a statistically significant impact on PFS in univariate analysis was the achievement of at least VGPR pre-ASCT. However, in multivariate analysis, none of these parameters retained independent prognostic significance, likely due to the strong interdependence between these factors. Moreover, our study identified a direct correlation between aMRD status and OS within the follow-up period, with a median OS of 60 months for aMRD+ patients compared to not reached in those without graft contamination, *p* = 0.003. Similarly, poor OS outcomes were observed in patients who exhibited persistent bmMRD+, both before and after transplant, in conjunction with graft contamination (aMRD+).

These findings suggest that aMRD assessment may serve as a valuable surrogate for residual disease burden at transplant and underscore the importance of effective induction therapy to achieve deep remission prior to ASCT. In conclusion, strategies aiming to optimise induction regimens—potentially through the use of anti-CD38 monoclonal antibodies—and MRD-guided treatment personalization could improve long-term outcomes in MM patients undergoing ASCT. Therefore, equivalent data are needed for patients treated with new standard of care regimens, such as dara-VTD or dara-VRD.

## 5. Conclusions

Our study highlights the critical importance of evaluating apheresis product contamination in MM patients undergoing ASCT. The presence of APCs in the graft was significantly associated with suboptimal responses to induction therapy and persistent bone marrow MRD positivity. Notably, graft contamination by APCs adversely affected the achievement of post-transplant bmMRD negativity, which should be the primary treatment goal for newly diagnosed MM patients receiving ASCT. This was further reflected in significantly reduced PFS and OS in patients with contaminated grafts.

These findings suggest that improving induction strategies to achieve deeper pre-transplant responses and implementing MRD-based risk stratification could enhance patient outcomes. The recent incorporation of anti-CD38 monoclonal antibodies into first-line treatment regimens in routine clinical practice may contribute to such improvements by enhancing the depth of response prior to transplantation. Importantly, patients who achieved MRD negativity before ASCT showed no evidence of graft contamination and experienced the most favourable survival outcomes, reinforcing the value of deep remission prior to stem cell collection and transplant.

## Figures and Tables

**Table 1:** Patients’ characteristics.

Characteristics	Patients (No. = 100)
**Sex** **—no. of pts**	
Male	51 (51%)
Female	49 (49%)
**Median age at diagnosis (range)**	59 (37–70)
**Cytogenetic risk—no. of pts (%)**	
Standard risk	68 (68%)
High risk	26 (26%)
- del(17p)	4
- t(4;14)	13
- t(14;16)	1
- gain(1q)/amp(1q)	15
NA	6 (6%)
**International Staging System (ISS)—no. of pts (%)**	
-I	31 (31%)
-II	23 (23%)
-III	20 (20%)
NA	26 (26%)
**Revised ISS (R-ISS)—no. of pts (%)**	
-I	26 (26%)
-II	40 (40%)
-III	6 (6%)
NA	28 (28%)
**Induction treatment**	
VTD	96 (96%)
PAD	4 (4%)
**ASCT—no. of patients (%)**	
Performed	81 (81%)
Not performed	19 (19%)

ASCT = autologous stem cell transplantation; NA = not available; PAD = bortezomib, doxorubicin and dexamethasone; VTD = bortezomib, thalidomide and dexamethasone.

**Table 2:** Impact on PFS of aMRD, and pre- and post-transplant responses.

Risk Factors		No. pts	Median PFS (month)	PFS, 24 month (%)	*p.*
**Pre-ASCT response** **(** ***n*** **° = 81)**	≥VGPR and aMRD−	59	N.R.	96%	<0.001
	≥VGPR and aMRD+	7	47.6	86%	
	<VGPR and aMRD−	10	45.1	90%	
	<VGPR and aMRD+	5	33.9	90%	
**bmMRD pre-ASCT** **(** ***n*** **° = 81)**	bmMRD− and aMRD−	35	N.R.	100%	0.025
	bmMRD+ and aMRD+	12	38.6	97%	
	bmMRD+ and aMRD−	34	N.R.	94%	
**Post-ASCT response** **(** ***n*** **° = 81)**	≥CR and aMRD−	52	N.R.	98%	<0.001
	≥CR and aMRD+	8	47.7	99%	
	<CR and aMRD−	17	62.2	99%	
	<CR and aMRD+	4	33.9	99%	
**bmMRD post-ASCT** **(** ***n*** **° = 77)**	bmMRD− and aMRD−	52	N.R.	98%	<0.001
	bmMRD− and aMRD+	15	N.R.	100%	
	bmMRD+ and aMRD−	5	N.R.	100%	
	bmMRD+ and aMRD+	5	35	80%	

aMRD = minimal residual disease evaluated on apheresis sample; ASCT = autologous stem cell transplantation; bmMRD = minimal residual disease evaluated on bone marrow sample; CR = complete response; N.R. = not reached; PFS = progression-free survival; VGPR = very good partial response.

**Table 3:** Impact on OS of aMRD, and pre- and post-transplant responses.

Risk Factors		No. pts	Median OS (month)	OS, 48 month (%)	*p.*
**Pre-SCT response** **(** ***n*** **° = 81)**	≥VGPR and aMRD−	59	N.R.	95%	<0.001
	≥VGPR and aMRD+	7	N.R.	97%	
	<VGPR and aMRD−	10	N.R.	97%	
	<VGPR and aMRD+	5	57	90%	
**bmMRD pre-ASCT** **(** ***n*** **° = 81)**	bmMRD− and aMRD−	35	N.R.	94%	0.010
	bmMRD+ and aMRD+	12	60	90%	
	bmMRD+ and aMRD−	34	N.R.	95%	
**Post-ASCT response** **(** ***n*** **° = 81)**	≥CR and aMRD−	52	N.R.	92%	<0.001
	≥CR and aMRD+	8	N.R.	83%	
	<CR and aMRD−	17	N.R.	95%	
	<CR and aMRD+	4	58	95%	
**bmMRD post-ASCT** **(** ***n*** **° = 77)**	bmMRD− and aMRD−	52	N.R.	97%	0.012
	bmMRD− and aMRD+	5	N.R.	99%	
	bmMRD+ and aMRD−	15	N.R.	99%	
	bmMRD+ and aMRD+	5	60	80%	

aMRD = minimal residual disease evaluated on apheresis sample; ASCT = autologous stem cell transplantation; bmMRD = minimal residual disease evaluated on bone marrow sample; CR = complete response; N.R. = not reached; OS = overall survival; VGPR = very good partial response.

## Data Availability

Most data generated or analysed during this study are included in this published article. The complete datasets are available from the corresponding author upon reasonable request.
